# European Correspondence

**Published:** 1878-07

**Authors:** 


### EUROPEAN CORRESPONDENCE.

---

LONDON, May 25th, 1878.

*Editor Atlanta Medical and Surgical Journal:*

The last meeting, for this season, of the Harveian Society has just taken place, and the paper of the evening was by Prof. Lister, on "The effects of position upon local circulation." The Professor began by calling attention to the well-known fact, that if the hand is elevated above the head, the blood recedes from the limb; and the question arose as to the cause of this phenomenon. Was it gravitation pure and simple, or was some action of the vaso motor nerve concerned therein? He had performed two experiments to determine this point—vivisections. (The Professor was particular in saying that he had given the animals chloroform). In the first, an artery in the leg of a horse near the hoof was laid bare, and its size carefully measured with a callipers, under four conditions: first, with the legs dependent; second, with the legs horizontal, the animal lying on its side; third, with the legs perpendicularly upwards, the animal lying on its back; fourth, the piece of artery was cut out and another measurement taken. Drawings of these to a scale were shown, and it was demonstrated that the area of the artery in the third position was not one-third that of the first, and very little larger than that of the excised vessel. The same experiments made upon the femoral artery of a heifer gave similar results.

A man was next brought forward, whose arm being held perpendicularly upwards for some minutes, became blanched and bloodless; not only this, but the muscles of the shoulder showed evidence of the same action. An elastic bandage was rapidly and tightly placed round the limb, close to the shoulder, and the hand was allowed to hang down for eight minutes; but at the end of this time it presented the same appearance as when first the bandage was applied. The arm being now elevated, the bandage was removed, and in a few minutes the circulation was re-established, not only in its original quantity, but it was absolutely increased, as shown by the heightened color when compared with the other hand, and the course of the returning circulation could be plainly traced in the limb.

Prof. Lister stated that he had employed this method as a substitute for Esmarch's bandage, and with most favorable results, as it avoids all danger of over-compression; and further, there is no possibility of forcing poisonous fluids from the diseased extremities into the adjacent tissues. Moreover, the small quantity of blood left in the limb, although practically unimportant, enabled the vessels to be more easily discovered and tied, and from this it resulted that secondary hemorrhage, so common with Esmarch's bandage, did not occur with this system.

These are the facts and the practical application thereof, and now comes the theory. The Professor considered that the laws of gravitation and of hydraulics, heretofore invoked to explain these phenomena, were totally inadequate for that purpose. That the power of the heart was sufficient to drive the blood into the upraised extremety, was proved by the blood being driven into the uplifted arm upon the removal of the bandage; and the heart of a horse was found to be sufficiently powerful to raise a column of blood over eight feet high, which was more than double the distance from the hoof to the heart. It could not be the increased weight of the column, for the artery, at the lowest point, was found to be diminished in calibre as well as the vessel at the highest point; nor could the blanching of the muscles of the shoulder, and the subsequent great increase of vascularity in the previously blanched arm, be accounted for by this hypothesis. It might be suggested that the veins acted as a support, but it would be found that an easily compressible tube as a vein or piece of intestine could not act as a syphon.

Prof. Lister then put forward the theory that the action was due to the vaso-motor nerves, and illustrated his meaning by referring to the effect produced by plunging the hand into a vessel of ice water and then removing it—the first being followed by contraction of the vessels, which was succeeded by increased vascularity of the part. A similar phenomenon was observed after ligating an artery for aneurism, say the femoral, in which case the foot became cold, but subsequently the heat and redness were abnormally increased.

Other illustrations were brought forward, but these will suffice to give a general idea of Prof. Lister's theory.

In this day of more complicated invention, this already well-known system has been neglected as a means for performing bloodless operations, and surgeons will do well to act upon the timely and valuable suggestion.

A couple of days later I saw Prof. Lister perform a lithotomy, about which there was nothing remarkable but that he laid great stress on the value of thoroughly sponging out the wound with a solution of chloride of zinc, and not carbolic acid, as I suspected; but from the remarks of the Professor, I am led to conjecture that this choice is the result of clincal experience.

R. J. Nunn.

---

London, June 1, 1878.

The most striking feature to a medical visitor to London is the utter absence of medical organization. True, each great school or hospital is perfect in and within itself, but then when the medical material of this great metropolis is divided among a score or more of general hospitals, the facility to the student equals only that of a city of two hundred thousand inhabitants; and in addition to this, there exists another drawback in the immense draughts of cases to maintain special hospitals, of which there are many—fine institutions, unquestionably, but practically so much lost to the general student. Finally, to increase the difficulty, the operating hour is about the same in each of these institutions, i. e. about 1 or 2 o'clock, P.M., so that, practically, it is impossible to visit more than one hospital daily. But even if the hours were otherwise arranged, the immense distance at which they are situated from one another would still preclude the possibility of doing much more. This is most inconvenient, most unsatisfactory, and results from the extraordinary erratic manner in which too many things are done here. Hospitals spring up where they are not needed—through the short-sighted benevolence of misguided philanthropists or the energy of an aspiring physician—and the result is not only a fearful waste of medical opportunities, but frightful increase of expense. Here this condition of things will probably always continue. Personal ambition and other conflicting interests will ever preclude the possibility of any adjustment. The evil exists, and exist it will; but with us it need not be created, although there are syptoms of the same or a similar condition springing into existence in some of our larger cities.

I would advise the medical rambler to drop in at the Royal Orthopedic Hospital, on Oxford street, at 2 P.M., where twenty or thirty tenotomies of various kinds are usually to be performed, besides other operations in this specialty.

Contracting cicatrices are treated in this hospital by passing a stout wire through the base of the cicatrix. A head on one end and a nut screwed on the other, retains this wire in its place, where it is allowed to remain until the track heals perfectly, and then the cicatrix is divided down to the wire, which is removed; extension is then adopted, and the results are said to be most favorable.

Daily, at 11 A.M., quantities of operations and treatments for various affections of the eye are to be seen at the Royal Ophthalmic Hospital, probably the largest institution of this kind in London. Here I saw some nævi treated by electrolysis, and an effort made to extirpate some eyelashes by the same means, which remind me of some improvements—as I consider them—that I adopted some years ago. In using electrolysis, I substitute a wire of suitable metal for the needle. This wire is insulated except near the point. To introduce the wire, first thrust in a fine trocar with its canula, then withdraw the trocar, leaving the canula; next push the insulated wire through the canula, and finally remove the canula while keeping the wire "in situ." By this simple expedient the skin is entirely relieved from electrolytic action. I have tried an insulated canula, but the wire seems to me preferable, but by either method the sharpness of the perforating instrument can be preserved uninjured by galvanic action, while electrolysis goes on within the tissues, the skin remaining unharmed.

When destroying a hair bulb, i. e. eyelash, by galvanism, it has been my practice first to pull out the hair, then thrust in a fine needle as near the track of the hair as possible, and apply the current, the idea being to seal up the track by the inflammation set up. This plan I have frequently used successfully, and it differs from, and is, I think, better than the method I have seen adopted here, in which the hair is not removed.

The apparatus of Mr. Clover for the administration of ether appears to be in general favor here. Its peculiarities consist in the re-inhilation of the same etherized air, and the regulation of the proportion of ether and of air. Its advantages are: diminution of the amount of ether required to the extent of $\frac{3}{4}$ or even $\frac{7}{8}$, shortening the time required to produce anæsthesia, and diminishing the struggling and nausea to a remarkable extent.

Dr. B. W. Richardson has just terminated the course of "Cantor Lectures" for this season. The subject chosen was the "putrefaction of meat," and the object appeared to be the discovery of some means by which meat could be preserved fresh. The whole matter was handled with that clearness and originality for which the lecturer is remarkable; and he illustrated the lecture with a hundred or more experiments. The novel theory was here advanced that the putrefaction of meat depends on the decomposition of the water it contains; and hence, in seeking for some preventive the necessary qualities it should possess are, that it should not change the appearance of the flesh, that it should fix the water in the flesh, that it should not change the flavor of the meat, and that it should be decomposed by the heat used in cooking the meat, while it should be unaffected by the heat of the atmosphere. Among the many substances experimented on, "Formate of Soda" was found to approach most nearly to the required qualities. About one or two per cent. was found to be sufficient to keep meat perfectly sweet for nearly two months, and the flesh when cooked was found to possess its original flavor; but this salt, although not affecting the color, did not preserve it, and so the vessel containing the meat was filled with washed coke smoke, (carbonic oxide) which perfectly preserves the red color of the flesh.

The lecturer tried the effect of forming salts in the substances of the flesh by passing different gases successively into the vessel containing the meat, with satisfactory results, and he suggests this as being a most promising field of enquiry. R. J. Nunn.

---

